# Profile and postrehabilitation outcome of patients with vertigo in a clinic in Southern Santa Catarina

**DOI:** 10.1055/s-0045-1811175

**Published:** 2025-08-31

**Authors:** Emanuela Hannoff Pilon, Vitor Benincá-Fernandes, Letícia Fernandes, Karina Rossa, Tatiana Pizzolotto Bruch

**Affiliations:** 1 Universidade do Extremo Sul Catarinense, Faculdade de Medicina, Criciúma SC, Brazil.; 2 Universidade do Extremo Sul Catarinense, Faculdade de Fisioterapia, Criciúma SC, Brazil.; 3 Universidade do Extremo Sul Catarinense, Faculdade de Matemática, Criciúma SC, Brazil.

**Keywords:** Epidemiology, Health Profile, Rehabilitation, Vertigo

## Abstract

**Background:**

For the purposes of the present study, we define *vertigo* as the sensation of rotation of the environment or of the person moving. The demand for care for this complaint has been increasing; therefore, it is important to know the profile of patients treated with this complaint, the causes, the comorbidities, the associated factors, and the response of these individuals to rehabilitation.

**Objective:**

To analyze the epidemiological profile of patients reporting vertigo and their outcome after physiotherapeutic rehabilitation.

**Methods:**

We conducted a descriptive cross-sectional study of secondary data analysis. The sample was collected through a census in a specialized clinic in the south of the state of Santa Catarina, Brazil. Individuals were evaluated through a questionnaire developed by the authors, which covered the patient's demographic and clinical data. The statistical analysis was performed using the IBM SPSS Statistics for Windows (IBM Corp.) software, version 23.0.

**Results:**

A total of 200 medical records were evaluated, 5 of which were excluded due to non-adherence to treatment, totaling a sample of 195 patients. Their mean age was of 51.18 ± 16.84 years, 62.6% were female subjects, and benign paroxysmal positional vertigo (BPPV) was the most prevalent cause (55.9%), with associated symptoms such as tinnitus (30.8%) and depression and/or anxiety (29.2%). Of the 195 patients, 193 responded positively to the vestibular rehabilitation treatment, and the vertigo of 58% improved after 1 session.

**Conclusion:**

The population older than 50 years of age is the most affected by vertigo, especially women, with BPPV as the main cause and associated symptoms such as tinnitus, depression, and anxiety. Most patients improved after the first vestibular rehabilitation session.

## INTRODUCTION

Vertigo is a symptom of vestibular dysfunction represented by a sensation of movement, 1 usually of the rotational type, as if the body or the environment were moving. This sensation can be confused with dizziness, whereas vertigo is a type of dizziness. 2

Vertigo can be a symptom of peripheral or central vestibular diseases, with a lifetime prevalence of up to 10% 3 and an annual incidence of 1.4%, 4 although it may still be underestimated when performing an uncertain examination of the vestibular and oculomotor system. 5 It is a common complaint in emergency and primary care, 1 in addition to causing significant restrictions in activities of daily living and quality of life. 6

Information received from the vestibular, visual, and somatosensory systems helps maintain balance. In addition, there is the membranous labyrinth, which contains otolithic organs (the saccule and utricle) and semicircular canals, which, when activated, enable us to understand various physical movements in everyday life. 7 Vestibular disorders present symptoms of vertigo, most frequently, in decreasing order, benign paroxysmal positional vertigo (BPPV), vestibular migraine, Ménière's disease, and vestibular neuritis. 8 Many elderly people with vestibular disorders have a history of many depressive and/or anxious symptoms, with a poor quality of life and persistent negative feelings related to sadness. 9

The diagnosis and treatment of the cause of vertigo vary according to its etiology and site of involvement, with the diagnosis being established by physical examination, positioning maneuvers or imaging tests. 10 11 12 The main and one of the most treatable causes of vertigo is BPPV, in which one or more of the semicircular canals are abnormally stimulated by otoconia that are dislodged from the otolith organs. 13 The benign disease has a prevalence ranging from 10.7 to 64 cases per 100 thousand inhabitants, in addition to a lifetime prevalence estimated at 3.2% in women, 1.6% in men, and 2.4% overall. 14

Vestibular rehabilitation is the main treatment, and it has been proven useful in resolving symptoms and improving body balance by restoring the sense of balance in people suffering from vestibular dysfunction. 15 16 In the case of BPPV, rehabilitation is based on repetitive exercises, performed through repositioning maneuvers. 17 Treatment of hypofunction uses vestibular stimulation to generate compensation, while movement sensitivity treatments use habituation to reduce or eliminate the provocative movements that cause dizziness. 18 However, BPPV is not the only cause of vertigo, and, for other diseases, systematic therapeutic exercises and functional training on the ground, virtual reality, adaptations to the home and the work environment can be performed. 19 When dealing with BPPV, the main cause of vertigo found in this study, most patients require one or two consultations for symptom relief and functional recovery, although some people may require several consultations for resolution. 16 20

Vertigo is a very common symptom that causes many physical and psychological difficulties for patients, in addition to increasing the cost of medical care. Therefore, knowing the epidemiological profile of the patients and the main causes can provide valuable information for the development of prevention strategies (in addition to generating awareness of the symptom among the population), as well as health education and evaluations of the impact of vestibular rehabilitation in the treatment of this clinical condition. The objective of the present study was to analyze the epidemiological profile of patients treated for vertigo and their postrehabilitation outcomes.

## METHODS

### Study design

We conducted a descriptive cross-sectional study, with collection of secondary data from medical records of patients with complaints of vertigo, treated from 2021 to 2023 at a clinic specialized in vestibular rehabilitation.

### Ethical aspects

The project was approved by the Ethics in Research Committee of Universidade do Extremo Sul Catarinense (UNESC), under opinion number 6.438.092.

### Study population

The medical records of 200 patients with complaints of vertigo, of all age groups, treated from 2021 to 2023 at a private clinic specialized in vestibular rehabilitation, in the city of Criciúma, state of Santa Catarina, Brazil, were evaluated through a census collection.

### Exclusion criteria

Patients with complaints of vertigo who did not adhere to the rehabilitation treatment.

### Data collection

Information on sex, age, cause of vertigo, comorbidities, associated symptoms, medication use, treatment adherence, and improvement were collected from the medical records. Improvement was assessed through patient feedback, reported to the physiotherapist or, when possible, in a face-to-face consultation to perform positional, ocular, and balance tests. The interventions performed were repositioning maneuvers, in which the patient underwent one session and returned or not, depending on the symptoms. Since the study was conducted in a vestibular rehabilitation clinic, no treatment medications were used.

### Statistical analysis

The statistical analysis was performed using the IBM SPSS Statistics for Windows (IBM Corp.) software, version 23.0. The qualitative variables were expressed as frequencies and percentages, and the quantitative variables were expressed as mean and standard deviation values.

## RESULTS

A total of 200 medical records of individuals who underwent vestibular rehabilitation with complaints of vertigo between 2021 and 2023 were evaluated, and 5 patients who did not adhere to treatment were excluded from the data collection, resulting in a total sample of 195 patients. The mean age of the sample was of 51.18 ± 16.84 years, and 62.6% were female patients, as illustrated in Table 1 .

**Table 1:** Profile of the study sample

		n = 195
Mean age (years)		51.18 ± 16.84
Sex: n (%)	Female	122 (62.6)
	Male	73 (37.4)

Table 2 shows the main causes of vertigo found. In total, 109 (55.9%) patients presented BPPV as the cause of vertigo. Comorbidities and/or associated symptoms are described in Table 3 , and patients may present more than one comorbidity. The most prevalent finding was tinnitus (30.8%). In addition, 29.2% of the patients reported having depression and/or anxiety, and 20.5%, systemic arterial hypertension (SAH). When analyzing each individual, 30.8% had tinnitus, 29.2%, depression and/or anxiety, 20.5%, hypertension, 12.3%, hypercholesterolemia, 7.7%, type-2 diabetes mellitus (DM2), 7.7%, hypovitaminosis D, and 4.1%, history of stroke.

**Table 2:** Causes of vertigo among the study sample

Cause: n (%)	n = 195
Benign paroxysmal positional vertigo	109 (55.9)
Vestibular neuritis	4 (2.1)
Ménière's disease	2 (1.0)
Other causes	48 (24.6)
No defined cause	32 (16.4)

**Table 3:** Comorbidities and/or associated symptoms of the study sample

Comorbidity and/or symptom: n (%)	n = 195*
Tinnitus	60 (30.8)
Depression and/or anxiety	57 (29.2)
Systemic arterial hypertension	40 (20.5)
Hypercholesterolemia	24 (12.3)
Diabetes mellitus	15 (7.7)
Hypovitaminosis D	15 (7.7)
Stroke history	8 (4.1)
Other comorbidity	84 (43.1)

Note: *Patients may present more than one comorbidity.

The patient outcomes are described in Table 4 . Of the 195 participants, 193 (99%) showed improvement in vertigo after treatment, with 58% improving after just 1 session. Of these 58%, although more than half had BPPV as the cause, other causes may also be involved, and the number of sessions and response to treatment is individual, regardless of the cause.

**Table 4:** Outcome of rehabilitation among the study sample

		n = 195: n (%)
Adherence to treatment		195 (100)
Improvement after treatment		193 (99)
Improvement after how many sessions	1	122 (58)
	2	45 (23.3)
	3	19 (9.8)
	≥ 4	17 (8.8)

## DISCUSSION

The current study aimed to evaluate the epidemiological profile of patients treated for vertigo and their postrehabilitation outcomes. This assessment was performed by evaluating demographic data, such as sex, age and the cause, crossing the data and evaluating each etiology, as we also sought to evaluate the main etiologies. We observed that the sample was mostly composed of female patients, with an average age over 50 years, and that 99% of the patients showed improvement, 58% of whom, after the first session.

When comparing this with the age profile found in the research, a study 4 states that 15 to 20% of the adult population is affected by vertigo and dizziness. The predominance of female subjects is in agreement with other studies, 21 22 which report that women seek medical care more and that metabolic and hormonal changes in women may influence the greater occurrence of vestibular disorders. When analyzing the various causes of vertigo, BPPV is the most prevalent, 23 being the etiology responsible for 20 to 53% of patients referred to specialized clinics. 24 These values found in the literature are similar to those of the present study, with BPPV being the cause responsible for 55.9% of the cases.

In addition, the presence of comorbidities or associated symptoms is important in relation to vertigo, with tinnitus being closely related, as already demonstrated by a Brazilian study, 25 which analyzed patients with an average age of 64 years, in which 46% presented vertigo and concomitant tinnitus, representing a value slightly higher than the percentage found in the present study (30.8%). In addition, when restricting the tinnitus research to the elderly population, another Brazilian study 26 which analyzed subjects with an average age of 73.86 years found the presence of tinnitus in 62% of the elderly people with vertigo.

When analyzing the psychiatric profile of the present study, 29.2% presented depression or anxiety. This data corresponds to that found in the literature: in a study from São Paulo, 9 29.5% of the elderly subjects analyzed presented symptoms of generalized anxiety disorder. Vertigo complicated by psychological problems is still a complex issue, and research 27 addresses a relationship of overlapping pathways in the central nervous system that transmit emotional and vestibular information. Depression is related to the formation of negative emotions, and this can worsen after repeated attacks of vertigo, which cause physical discomfort and affect the patient's quality of life. 28

In the current study, 20.5% of the patients presented SAH, and 7.7%, DM2, which corroborates studies that state that vestibular dysfunctions have more than 1 associated clinical alteration. 29 30 Studies 31 32 state that the presence of SAH brings a risk of recurrence of BPPV, due to vascular damage that leads to labyrinthine ischemia and detachment of the otoconia. Additionally, 7.7% of the patients in the current study presented hypovitaminosis D, and a randomized study 33 conducted between 2013 and 2017 showed that vitamin D supplementation may help prevent BPPV attacks.

The majority (99%) of the patients in the present study improved after treatment with vestibular rehabilitation exercises, confirming studies 32 33 34 that demonstrate increased functionality and improved quality of life. It cannot be stated that these patients who improved were in fact those with BPPV, since a general analysis of the data was performed, without a specific focus on any etiology. Vestibular rehabilitation exercises can be performed in several ways, as mentioned in the introduction, depending on the cause. 19 For causes such as neuritis, hypofunction, and vestibular migraine, exercises with platforms, vibrotactile repositioning, virtual reality, and neuromodulation are performed. 35 36 When the etiology is BPPV, specific maneuvers are used, such as Epley and Semont for the posterior canal or Lempert for the lateral canal. 35 36 Of these improvements, 58% occurred after the first treatment session and 23.3%, in the second session, corroborating studies 37 38 39 that showed that the degree of improvement varies among patients. This improvement in symptoms after performing the maneuvers confirms that the stimuli to the otoconia were removed and body balance was restored, which are the objectives of vestibular rehabilitation exercises. 37 38 40

The present study had limitations due to the use of secondary data obtained from medical records, which restricted the researchers to the information available and recorded at the time of care. In addition, it was not possible to perform more in-depth cross-referencing among the variables due to the lack of standardization or incomplete data, together with the limited time to write the current work.

For future research, we suggest conducting studies in which psychiatric factors and stroke with vertigo symptoms are evaluated. Furthermore, research can be performed to better understand the association between tinnitus and vertigo, as they are commonly associated symptoms, aiming for better therapy and potential means of prevention.

We conclude that the population in the southern region of Santa Catarina over 50 years of age is more affected by vertigo, mainly women, and they present associations with tinnitus, depression, and anxiety. The main cause found was BPPV, and most of the patients improved immediately after the first vestibular rehabilitation session.
